# Liver X Receptor Inverse Agonist SR9243 Suppresses Nonalcoholic Steatohepatitis Intrahepatic Inflammation and Fibrosis

**DOI:** 10.1155/2018/8071093

**Published:** 2018-02-18

**Authors:** Peng Huang, Benson Kaluba, Xiao-lin Jiang, Shi Chang, Xiao-feng Tang, Lin-feng Mao, Zhi-peng Zhang, Fei-zhou Huang

**Affiliations:** ^1^Department of General Surgery, The Third Xiangya Hospital, Central South University, No. 138 Tongzipo Road, Changsha 410013, China; ^2^Department of General Surgery, Xiangya Hospital, Central South University, No. 87 Xiangya Road, Changsha 410008, China; ^3^Department of Hepatobiliary Surgery, The First People's Hospital of Jingzhou, Yangtze University Health Science Center, No. 8 Hangkong Road, Jingzhou 434008, China

## Abstract

Abnormal metabolism of cholesterol may be a contributing factor in nonalcoholic steatohepatitis (NASH) pathogenesis. Accumulating evidence has shown that liver X receptor (LXR) is closely related to intrahepatic inflammation and fibrosis. In this study, we evaluated the effects of a novel liver-specific LXR inverse agonist, SR9243, on antifibrosis in NASH mice. A high-cholesterol diet was employed to induce NASH in BALB/c mice by either carbon tetrachloride (CCL4) administration or bile-duct ligation (BDL). Once NASH was induced, mice were treated with SR9243 for one month by intraperitoneal (i.p.) injection. Liver tissues were collected to determine the degree of fibrosis and intrahepatic inflammation via pathological examination and QPCR; serum was collected to analyze the plasma lipid levels and liver function by clinical biochemistry. The mice developed hepatic steatosis, severe hepatic inflammation, and fibrosis by BDL or CCL4. Treatment with SR9243 significantly reduced the severity of hepatic inflammation and ameliorated hepatic fibrosis; simultaneously, body weight, serum glucose, and plasma lipid levels were controlled effectively. Our data demonstrate that SR9243 exerts an antifibrotic and anti-inflammatory effect in NASH mice; hence these findings highly suggest that LXR inverse agonist could be therapeutically important in NASH treatment.

## 1. Introduction

Nonalcoholic steatohepatitis (NASH) is considered as leading cause of hepatitis nonviral liver cirrhosis and hepatocellular carcinoma [1, 2]. In the development of NASH, nonalcoholic fatty liver disease (NAFLD) is the first step and is characterized by hepatic steatosis which is caused by an imbalance between fat/influx of energy and utilization [3, 4]. Since accumulating evidences have proved that efficiency in both lipid transport and delivery seems to be a crucial factor in transitioning from hepatic steatosis to NASH, high-calorie diets with excessive fats and carbohydrates can cause this imbalance leading to NAFLD and in some cases progression to NASH; in addition, intrahepatic cholestasis caused by biliary obstruction can also lead to NASH [5, 6]. Currently, there are no established treatment interventions for NASH; however, some new agents have emerged as potential therapeutic targets that can either activate or inhibit nuclear receptor signaling [6]. Liver X receptors (LXRs) control cholesterol and lipid metabolism via regulating gene networks as members of a super family of nuclear hormone receptors and they include two other homologous but different isoforms (LXR*α* and LXR*β*) [7]. Previous studies have proved that LXR*α* is highly expressed in kidney, liver, intestines, and adipose tissue while LXR*β* is expressed widespreadly throughout the body [8]. The potential of LXR as a therapeutic target in the pathogenesis of metabolic diseases by regulating metabolic and inflammatory pathways has recently been realized [9]. Known synthetic LXR agonists like GW3965 and T0901317 have previously been reported to reduce neuroinflammation, limit inflammation, attenuate myocardial hypertrophy, prevent atherosclerosis, and reduce ischemia/reperfusion injury [10].

LXR agonists exhibit their antitumor activity by significantly lowering intracellular cholesterol levels in cancer cells [11–13]. Moreover, the LXR inverse agonist, like SR9238, showed well antifibrosis effect [14]. Recently, some studies have brought to light the emerging role of LXR in tumor metabolism, immune evasion, and also progression. LXRs also participate in receptor-mediated downregulation of lipogenic and glycolytic enzyme expression, for which LXR inverse agonists can be more better selective therapeutic agents than targeted enzyme inhibition to disrupt the Warburg effect and lipogenesis [15]. SR9243, one of novel LXR inverse agonists, displayed safety in noncancer cells and tissues and may be an important part in the mechanism of action in lipogenic and glycolytic gene suppression mediated by LXR [16].

In this study, we sought to determine if such a therapeutic agent would have efficacy in reduction of both fibrosis and inflammation in a mouse model of NASH; we therefore used experimental models involving administration of high-cholesterol (HC) diets to mice in which liver fibrosis was induced by either bile-duct ligation (BDL) or carbon tetrachloride (CCl4) intoxication.

## 2. Materials and Methods

### 2.1. Animal and Animal Care

Seventy-two 8-week-old wild-type BALB/c healthy male mice were used for the animal model and kept in a special pathogen-free environment where temperatures were maintained at 20–25°C and humidity at 50–70% [17]. The mice were acclimatized to this new environment for two weeks prior to commencing the experiments. Laboratory ethical requirements for animal care were observed during the experiments.

### 2.2. Animal Models

The mice were randomly separated into twelve experimental groups (*n* = 6 per group), and were fed either a high-cholesterol (HC) (1% wt/wt) diet (TD 92181) or a control diet (Teklad no. 7001; Harlan Teklad, Madison, WI) for 4 weeks, and then either underwent BDL for 3 weeks or were given CCl4 at a dose of 5 *μ*L (10% CCl4 in corn oil)/g body weight, by intraperitoneal injection twice a week for 4 weeks.

After six weeks, the mice continued on the NASH diet and were treated with 30 mg/kg SR9243 q.d. i.p. in 10% DMSO/10% Tween-80/80% water or vehicle for 30 days during which food intake and body weight were monitored daily. At the termination of dosing, blood was collected through the eyeball method and analyzed using clinical biochemistry and ELISA. Liver tissues were collected and weighed, and a portion was immediately freshly frozen in liquid nitrogen for RNA analysis. The rest were placed in 10% neutral buffered formalin for histology.

### 2.3. Biochemical and Histologic Analysis

Serum concentrations of ALT, TGs, glucose, and cholesterol were determined using a Fuji Dry-Chem 5500 (Fuji Film, Tokyo, Japan). Concentrations of liver hydroxylproline and hepatic TG content were measured as described in a previous report [18]. After being fixed with 4% paraformaldehyde, liver tissues were embedded in paraffin and then stained with H&E and a Masson trichrome solution. Liver tissues were also frozen in liquid nitrogen and stored at −80°C until when needed for either protein or RNA analysis [19]. RNA was isolated from liver tissues and QPCR was used for analysis as described previously [20].

### 2.4. Statistical Analysis

All the data are expressed as the means ± standard errors of the means. Statistical analyses were performed using the unpaired Student's *t*-test or one-way analysis of variance (*P* < 0.05 was considered significant).

## 3. Results

### 3.1. SR9243 Significantly Decreased Liver Fibrosis Induced by BDL and CCL4

**Figure 1:**
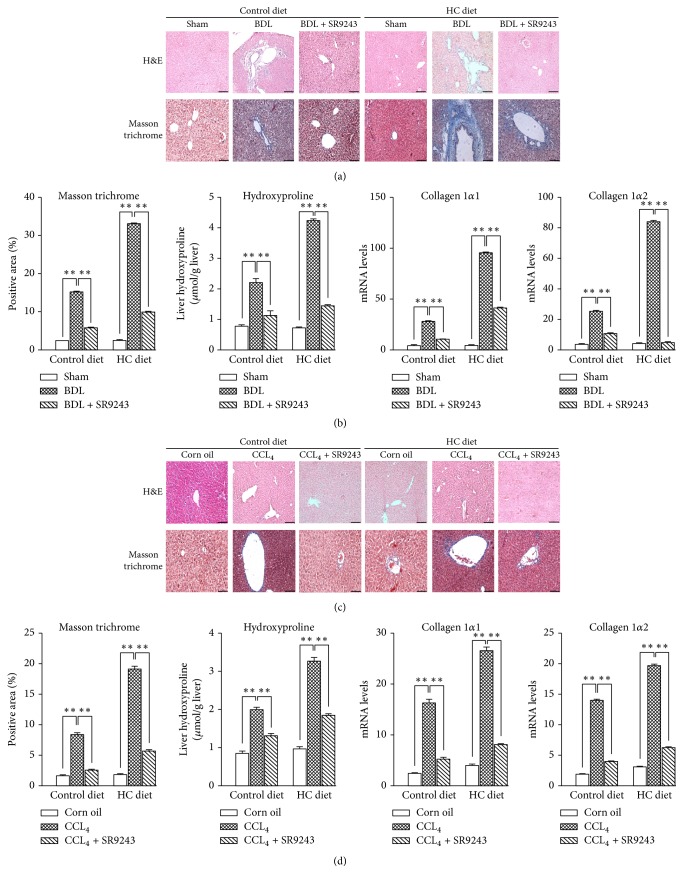
Effects of SR9243 on liver fibrosis induced by BDL or CCL4 treatment. After being fed a control or HC diet for 4 weeks, BALB/c mice were subjected to (a, b) 3-week BDL or (c, d) CCl4 treatment twice a week for 4 weeks to induce NASH model (*n* = 6/group). ((a) and (c)) H&E-stained sections and Masson trichrome-stained sections in representative liver samples. ((b) and (d)) Quantification of Masson trichrome staining, liver hydroxyproline concentrations, and hepatic expression of collagen 1*α*1 and collagen 1*α*2. ^*∗∗*^*P* < 0.01.

There are reports that high-cholesterol diet is sufficient to induce a NASH phenotype that correlates to human disease pathology [21]. Based on this diet, we induced chemical damage-induced NASH and biliary NASH, and on the NASH model we examined the potential efficacy of SR9243. As shown by Masson trichrome staining of liver tissue (Figure 1), we clearly observed from the pathological point of view, BDL significantly exacerbated liver fibrosis in both the control diet and HC diet group. However, the degree of liver fibrosis in the HC diet group was more significant as compared to the control diet group. The mRNA expressions of hydroxyproline, collagen 1*α*1, and collagen 1*α*2 were significantly promoted as a result of liver fibrosis induced by BDL and this was seen more clearly in the HC diet group than in the control group.

After treatment with SR9243, we evaluated the effect of SR9243 on hepatic fibrosis by the same test. The results showed that SR9243 significantly inhibited liver fibrosis in mice. The results were the same in both the normal diet and the HC-fed NASH groups.

In a similar manner to the BDL model, the murine CCl4 model of liver fibrosis showed a significant progression of liver fibrosis in the HC diet group versus control, and SR9243 was also effective in inhibiting CCl4-induced liver fibrosis.

### 3.2. Effects of SR9243 on Mouse Body Weight, Insulin Levels, and Blood Glucose

**Figure 2:**
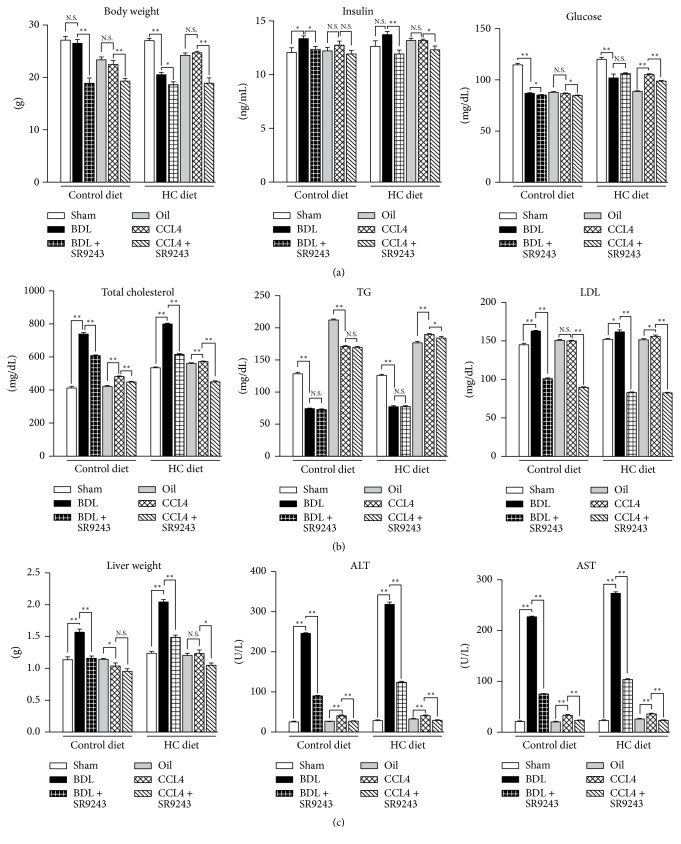
Effects of SR9243 on hepatocyte injury induced by BDL or CCL4 treatment. (a) Fasting insulin levels are shown in the middle panel; glucose levels are illustrated in the right panel. (b) Total cholesterol, LDL, and plasma triglycerides were determined from mouse plasma samples at the termination of the experiment. (c) Liver enzyme levels changed during the treatment. ^*∗*^*P* < 0.05 and ^*∗∗*^*P* < 0.01.

Although we noted no alterations in food intake during the treatment period, we did observe a significant decrease in body weight after the treatment (Figure 2). We found that CCL4 did not cause significant changes in body weight in both normal and HC diet groups. However, BDL decreased body weight in HC-treated mice, while SR9243 significantly reduced body weight in all interventions, the reason may be that the degree of ascites in the treatment group was lower than that in the control group.

We further measured the level of insulin and found that BDL increased insulin levels in the normal diet group, but not so obvious in the HC diet group; effects of CCL4 on insulin levels were not obvious; however, SR9243 has a certain inhibitory effect on insulin. The results of blood glucose test showed that SR9243 controlled the blood sugar level in the intervention group, which was more obvious in CCL4 model under HC diet (Figure 2).

### 3.3. Effect of SR9243 on Blood Lipid Levels

We observed that both BDL and CCL4 caused an increase in total cholesterol, which was more pronounced in the HC diet (Figure 2). SR9243 significantly inhibited mouse total cholesterol levels. We also found that BDL decreased TG levels, while CCL4 showed the opposite results; meanwhile, the effect of SR9243 on TG was not obvious. BDL and CCL4 cause LDL levels to rise, but this phenomenon is significantly inhibited by SR9243.

### 3.4. Effects of SR9243 on Liver Functions

To understand the changes in liver functions, we measured the weight of the liver and the indicators of liver functions (Figure 2). Except for the increase in liver weight caused by BDL, the weights in other groups did not change significantly compared with the control group. From our test results, BDL and CCL4 can cause a significant increase in ALT and AST, and these increased liver enzymes can be inhibited by SR9243.

### 3.5. Effect of SR9243 to Reduce Hepatic Inflammation

**Figure 3:**
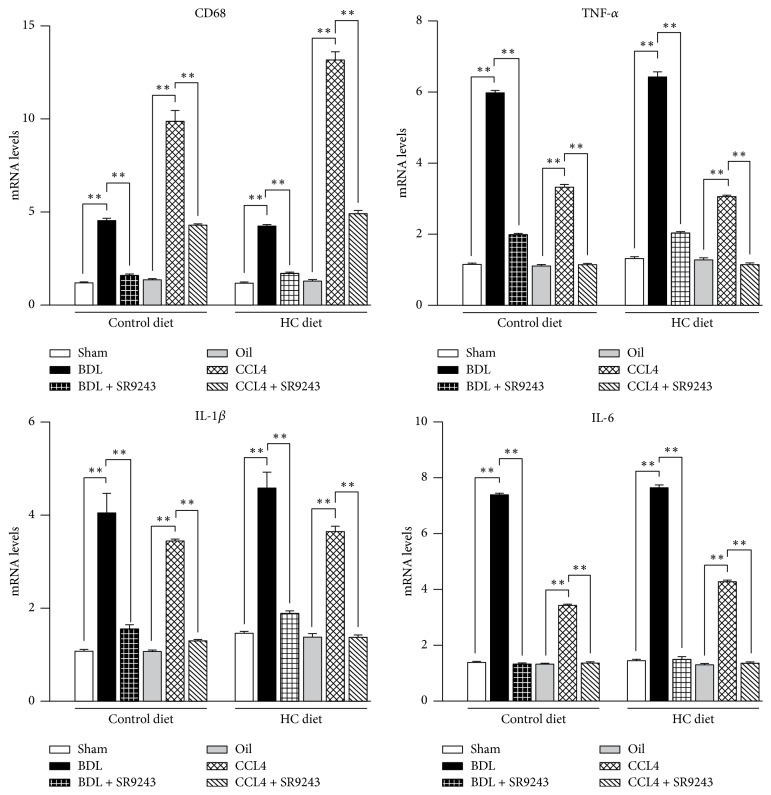
Effects of SR9243 on hepatic proinflammatory cytokines expression induced by BDL or CCL4 treatment. mRNA levels of CD68, TNF-*α*, IL-1*β*, and IL-6 were tested in liver tissues by QPCR, ^*∗∗*^*P* < 0.01.

To investigate whether SR9243 can reduce liver inflammation caused by NASH, we detected the mRNA expression of CD68, TNF-*α*, IL-1*β*, and IL-6 by QPCR (Figure 3). The trend of BDL and CCL4 increasing hepatic inflammatory injury was consistent. After treatment with SR9243, the levels of CD68, TNF-*α*, IL-1*β*, and IL-6 were significantly decreased compared with those in the control group.

## 4. Discussion

As a healing reaction to chronic liver injury, liver fibrosis poses a heavy burden on human health, and if the causative agent persists, it is likely to develop further into cirrhosis, liver failure, and even liver cancer, making it a common cause of death. A recent study reported a 20–50% mortality rate of end-stage liver disease resulting from fibrosis which consequently leads up to severe liver cirrhosis [22]. The development of liver fibrosis is promoted by chronic liver disease which is characterized by two distinct features: cell death and inflammation. NASH differs from simple steatosis due to the presence of inflammation, hepatocyte death, and also a varying degree of fibrosis. Various mechanisms have been proposed to support the transition from simple steatosis to NASH and NASH-related fibrosis, and this includes reactive oxygen species (ROS) whose production results from poorly regulated cholesterol and hepatic lipid metabolism [23]. This, in turn, recruits monocytes that are more potent proinflammatory agents than resident KCs and produce such cytokines like TNF-a and IL-1*β* which contribute to NASH and fibrosis development. There are no standard treatments for NASH which target on inflammation and fibrosis except for improved diets and weight loss.

Liver X receptors (LXRs) are nuclear, lipid-activated receptors whose important functions include lipogenesis, cholesterol transport, and anti-inflammatory signaling. During chronic liver injury, hepatic stellate cells are activated and facilitate the fibrotic response. Beaven et al. [24] reported that LXRs are among the most highly expressed nuclear receptors in stellate cells and that LXR signaling plays a role in regulating the expression of those genes which are linked to inflammation, metabolism, and fibrogenesis in primary cells. Accordingly, treatment with synthetic LXR activators might have a beneficial effect in models of fibrotic liver disease.

In this study, we demonstrated that continuous low dose (30 mg/kg) of LXR inverse agonist (SR9243) suppressed the liver fibrosis in chemical damage-induced NASH or biliary NASH mice models effectively; simultaneous, intrahepatic inflammatory factors have been relieved by varying degrees; this mechanism may be consistent with previous reports that showed LXR-mediated glycolytic and lipogenic gene suppression [16]. Griffett et al. [14] reported the same treatment effect of another LXR inverse agonist (SR9238). However, some studies recently published suggest that hepatotoxicity is one of the main side effects of currently available pan-LXR agonists [25]. Archer et al. [26] found that when ob/ob mice were treated for 5 weeks with the synthetic LXR agonist GW3965 (10 mg/kg), increased hepatic TG content and lipotoxicity were detected in mice due to activation effect on both LXR*α* and LXR*β*. Interestingly, we also observed that mice body weight and lipid levels were under control, while hepatotoxicity was not observed in this study, which revealed even more potential effects of inverse agonists to control liver fibrosis as compared to agonists.

Although the present results have provided obvious evidence of SR9243 in decreasing liver fibrosis in mice, lots of work should be done in the future. For it is challenging to obtain a LXR knockout mouse in a short time, we have not explained the pathway and multiple mechanisms involved in the process. And we plan to further examine the biosafety of SR9243 in an expanded animal model.

## 5. Conclusions

Our results show a significant therapeutic potential in treating NASH with LXR inverse agonist SR9243.
